# Measuring Oxygen Solubility in Amorphous and Semicrystalline Polyolefins Using Test Particle Insertion: A Comparative Study of Polyethylene and Isotactic Polypropylene

**DOI:** 10.1021/acs.jpcb.4c05106

**Published:** 2024-09-18

**Authors:** Nikolaos I. Sigalas, Stan A. T. van Kraaij, Fotis Venetsanos, Stefanos D. Anogiannakis, Doros N. Theodorou, Alexey V. Lyulin

**Affiliations:** † Soft Matter and Biological Physics Group, Department of Applied Physics, Technische Universiteit Eindhoven, 5600 MB Eindhoven, The Netherlands; ‡ Center for Computational Energy Research (CCER), P.O. Box 513, 5600 MB Eindhoven, The Netherlands; § Computational Materials Science and Engineering Group, School of Chemical Engineering, National Technical University of Athens (NTUA), GR-15780 Athens, Greece; ∥ DPI, P.O. Box 902, 5600 AX Eindhoven, The Netherlands

## Abstract

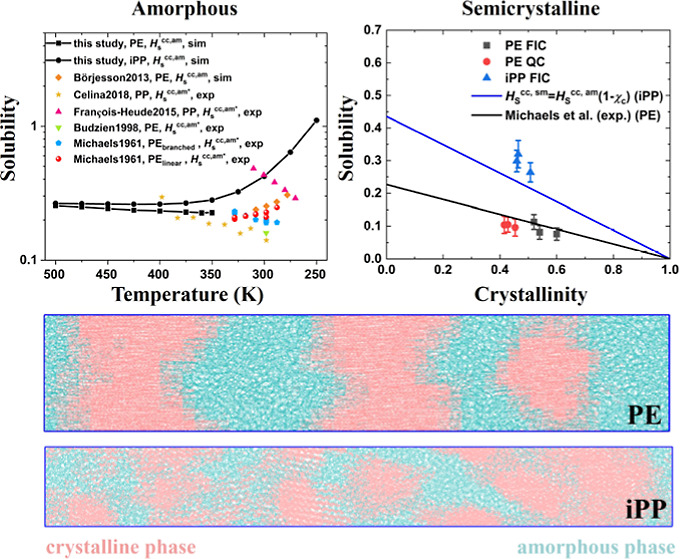

The test particle insertion method is used to study the solubility of oxygen in two commonly used polymers: polyethylene (PE) and isotactic polypropylene (iPP). Amorphous samples for both polymers were prepared by means of Monte Carlo and molecular dynamics simulations, and the oxygen solubility was measured across different temperatures. The solubility-temperature dependence for iPP proved to be nonmonotonic due to the interplay between binding and reorganizational enthalpy, while for PE, it appeared to be monotonic based on the available data in the studied temperature range. A broad comparison to experiments and simulations is included. Further oxygen insertions were also performed in semicrystalline PE and iPP samples at ambient temperature, and the obtained values were compared to a linear relationship which correlates the solubility in the purely amorphous phase with the solubility in the crystalline phase. The solubility of PE closely follows the linear relationship, while iPP exhibits some divergence. All the semicrystalline samples were previously annealed at elevated temperatures for long periods (a few μs), and a strong effect of annealing was observed on the structure and the solubility of iPP. A well-developed iPP lamellar structure emerged at longer annealing times, while PE develops that structure already in the early crystallization stages. The solubility of semicrystalline iPP samples with lamellar morphology exhibited better agreement with extrapolated solubility values of the amorphous state—the extrapolation was made using a linear relationship connecting solubility in the purely amorphous phase and solubility in mixed phases (amorphous and crystalline). Results on the correlation of the solubility with the local structural ordering are also present.

## 1 Introduction

Nowadays, the problem that plastic pollution poses to the environment is widely recognized. Packaging is one of the largest sectors of polymer production, and in recent years, significant efforts have been made to find solutions to tackle this issue. One promising route is the transition from multimaterial to monomaterial packaging. Most of the packaging used today is made of multilayered materials consisting of polymers with different chemical structures to address specific applications. Additionally, metal foils and other additives are used. Transitioning to monomaterial packaging means using a single polymer or polymers of similar chemical constitutions to create the desired material. An essential property of this material is to prevent gas permeation, as a significant amount of such packaging is used in the food industry.

The permeation of gases through polymeric membranes is a complicated process that is highly dependent on the morphology of the material. Permeation is essentially described by two stages: solvation and diffusion. Solvation involves the adsorption and absorption of gas molecules to the surface and into the bulk of the polymer, respectively. Diffusion is related to the transfer of molecules through the polymer bulk. Important penetrants to investigate include oxygen and water as they are crucial in determining whether a packaging material prevents oxidation and maintains the desired water content of the packaged product. Polymers for packaging are known to be semicrystalline in nature, meaning that they comprise both crystalline and amorphous phases. A linear relationship^1^ commonly describes the solubility of semicrystalline materials with respect to crystallinity
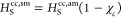
1where *H*_S_^cc,sm^ is the Henry’s law solubility constant measured in a semicrystalline polymer, *H*_S_^cc,am^ is the Henry’s law solubility constant measured in an amorphous polymer, and χ_c_ is the crystallinity. This relationship assumes that the crystalline phase is impermeable and that the amorphous phase is not affected by the presence of crystallites. These assumptions were first set by Michaels and co-workers^1^ in the 60s. However, there are instances where the crystalline phase can absorb gaseous molecules, such as in the case of syndiotactic polystyrene,^2^ where the crystalline phase is nanoporous. Another important consideration is that the densely packed crystal phase of certain polymers, e.g., monoclinic phase of polyethylene (PE), is not created instantly. Hikosaka et al.^3^ found experimentally that upon the crystallization of PE at elevated pressures, the formation of lamellar crystals passes through a mesomorphic hexagonal phase. This observation of the transient mesophase to a higher density stable form was also supported by Sirota.^4^ Thus, it is crucial to understand at which stage the morphology of the material is arrested. Up to date, experimental techniques are able to predict the fractions of this mesophase,^5^ and tailor its percentage based on the crystallization temperature. However, the structure and the stability of the mesophase, as well as the solubility of gaseous molecules in it, have not been explored in depth.

After the formation of the lamellar phase, the polymer has distinct crystalline and amorphous phases. The amorphous phase can be further distinguished in constrained and unconstrained, as it was highlighted by Chmelar et al.^6^ by analyzing ^1^H NMR and SAXS data from semicrystalline PE samples. The linear relation (eq 1) considers only the unconstrained or free phase. Thermodynamic models exist that account for all the three phases and can make decent predictions of the solubility of gases in semicrystalline polymers.^7,8^ Recently, several experimental cases where this relation fails have been discussed in a review paper by Marano et al.,^9^ primarily in polymers with high crystallinity content, where the percentage of the constrained amorphous phase is increased. The constrained amorphous phase exhibits solubility values lower than those of the unconstrained one.

There are several experimental and computational studies of the solubility of gases in polymers. Computational techniques such as the test particle insertion (TPI) method can be particularly useful.^10^ By applying the TPI method, Müller-Plathe studied the solvation of various gases in amorphous atactic polypropylene.^11^ Later, van der Vegt^12^ used the same technique to study the solvation of a series of gases in liquid dodecane and liquid PE, as well as for glassy polyvinyl chloride and poly(vinyl alcohol). Vergadou and Theodorou^13^ also studied the sorption of small molecules in glassy polymers. It should be noted that most computational studies focus on amorphous polymers, with fewer studies on semicrystalline polymers.^14^

In this study, we use the TPI method to investigate the solvation of oxygen in polymeric membranes. In Section 2, the protocols followed for preparing amorphous and semicrystalline PE and isotactic polypropylene (iPP) samples are described. In Section 3, we measure the solubility in purely amorphous PE and iPP at supercooled temperatures. In the same section, the solubility of oxygen is studied in samples of semicrystalline PE and iPP. The agreement with the linear relationship proposed by Michaels and Bixler^1^ is tested. Then, further investigation of the effect of annealing on the structure and solubility of iPP follows. Finally, further insights are provided on the correlation of the solubility with the local structure and local ordering.

## 2 Models and Methods

### 2.1 Model and Initial Configurations

An iPP melt of 20 chains with 2000 monomers per chain was simulated. An iPP monomer comprised a methyl group (CH_3_), a methylene group (CH_2_) and a methine group (CH) in the united atom representation where carbons and hydrogens are fused into one single united atom. A PE melt of 100 chains with 1000 carbon atoms per chain on average—the system had a polydispersity index of 1.08—was also simulated at the united atom level comprising a methylene group (CH_2_) and a methyl group (CH_3_). We used the TraPPE-UA force field that was initially developed by Martin and Siepmann^15^ and then modified by Pütz et al.^16^ for iPP to describe all the bonded and nonbonded interactions between different groups. The intramolecular nonbonded interactions are calculated only for united atoms that are four or more bonds apart. It should be noted that charges are not taken into account. TraPPE-UA also provides a description for molecular oxygen O_2_,^17^ which entails a three-site model. The model consists of an extra mass-less atom that resides at the exact center of the molecule and bears a nonzero charge to ensure that the molecule has a zero net charge; the two side oxygen atoms have a charge of −0.113 e each, while the mass-less atom charge equals to 0.226. Additionally, it should be noted that the mass-less atom does not interact via Lennard-Jones interactions. TraPPE-UA has been extensively used in crystallization studies of polymers, as was discussed in Ref (18) In all the simulations, a Verlet neighbor list for Lennard-Jones interactions was used with a cutoff distance for short-range neighbors *r*_list_ = 1.4 nm. In addition, a plain cutoff with an unmodified van der Waals potential was used with tail corrections for energy and pressure. The cutoff distances for van der Waals interactions were 1.2, 1.2, and 0.909 nm for iPP, O_2_, and PE, respectively. All the simulations were performed with Gromacs software package version 2020.1.^19^

### 2.2 Simulation Protocol for Preparing Amorphous iPP Samples

The first step was to create three initial configurations of the polypropylene with 100% meso dyads using the materials and process simulations (MAPS) platform developed by Scienomics.^20^ After that, the iPP melt was equilibrated at 500 K, above the melting temperature *T*_m_ of high molar mass iPP reported as 460 K.^21^ The equilibration was completed in three different steps: (1) an energy minimization using the steepest descent algorithm which converged when the maximum force was smaller than 10 kJ/(mol nm); (2) a simulation in the canonical ensemble (*NVT*), which lasted for 5 ns using the velocity rescaling thermostat with time constant τ(*T*) = 0.5 ps; and (3) a simulation in the *NPT* ensemble, which lasted for 100 ns using the Nosé–Hoover thermostat with time constant τ(*T*) = 0.5 ps and the Parrinello–Rahman barostat^22^ with time constant τ(*P*) = 5 ps. After the equilibration, each initial configuration was cooled below the melting temperature of iPP, equal to 460 K^21^, with a cooling rate of 1 K/ns to different temperatures. In total, 11 temperatures were studied at the supercooled and melt states without deformation, from 250 to 500 K with a step of 25 K. After the cooling, an *NPT* ensemble simulation was undertaken at the respective temperature for 100 ns. The last 50 ns were used for test particle insertions of oxygen in the amorphous iPP samples.

### 2.3 Simulation Protocol for Preparing Amorphous PE Samples

For the amorphous PE samples, we followed a different strategy. In the beginning, an initial melt configuration of purely amorphous PE was generated using the amorphous builder plugin of the MAPS platform.^20^ The generated configuration was purely monodisperse and consisted of 100 chains with 1000 methylene units per chain. Then, it was energy minimized and equilibrated using a home-built connectivity altering Monte Carlo (MC) algorithm^23^ at constant *T* = 450 K and *P* = 1 atm, producing a fully equilibrated trajectory of narrow polydisperse (*D* = 1.08) PE samples. From the fully equilibrated trajectory produced from our MC algorithm, we selected three independent configurations. Each one of them was brought by cooling down or heating up to eight different temperatures varying between 350 and 550 K using a rate of 1K/ns. The melting point of PE has been reported to be equal to 396.4 K.^36^ For each temperature, we let the systems relax for approximately 200 ns performing *NPT* MD simulations at constant atmospheric pressure and at the constant corresponding temperature. The last 50 ns were used for test particle insertions of oxygen in the amorphous PE samples.

### 2.4 Simulation Protocol for Preparing Semicrystalline iPP Samples

The three iPP samples equilibrated at 500 K were also used as starting points for preparing the semicrystalline samples. Specifically, each sample was cooled down to 410 K with a cooling rate of 1 K/ns and, then, was submitted to a simulation in the *Nė*_*xx*_*P*_*yy*_*P*_*zz*_*T* ensemble with a strain rate along the tensile direction of *e*_*xx*_ = 10^7^ s^–1^ and pressure along the perpendicular directions equal to *P*_*yy*_ = *P*_*zz*_ = 1 bar, which lasted for 300 ns. With the chosen strain rate, the simulation box was stretched up to four times its initial length along the *x* axis. After the stretching, an annealing simulation in the *NL*_*xx*_*P*_*yy*_*P*_*zz*_*T* ensemble was performed at the same temperature (410 K) which lasted for 5 μs. At the end of the annealing, the samples were cooled from 410 to 298 K with a cooling rate of 1 K/ns. Finally, another *NL*_*xx*_*P*_*yy*_*P*_*zz*_*T* ensemble simulation was performed at ambient temperature for 100 ns in total. During both the annealing and cooling stages, the length along the tensile direction was kept constant, and the pressure was set equal to the atmospheric pressure along the *y* and *z* directions.

### 2.5 Simulation Protocol for Preparing Semicrystalline PE Samples

The semicrystalline PE samples used in this study were prepared under two different conditions: (a) quiescent and (b) drawing conditions. For the samples prepared under quiescent conditions, three independent purely amorphous PE melt configurations were cooled down to the temperature of *T* = 340 K applying a constant, relatively fast cooling rate of 1 K/ns—each independent configuration was selected from the fully equilibrated connectivity altering MC trajectory we described in Section 2.3 at *T* = 450 K and *P* = 1 atm. Then, both temperature and pressure were kept constant at *T* = 340 K and *P* = 1 atm, respectively, and *NPT* MD simulations were performed while monitoring the evolution of the degree of crystallinity, χ_c_, until it reached a plateau value, after approximately 2.5 μ s. Then, the samples were cooled again to the desired final temperature of *T* = 298 K, applying the same cooling rate of 1 K/ns, and the system was left to relax under isobaric conditions for 200 ns. In the end, three independent PE semicrystalline samples were prepared at *T* = 298 K and *P* = 1 atm under quiescent conditions.

On the other hand, for the samples prepared under drawing conditions, the three independent purely amorphous PE melt configurations mentioned above were cooled to 365 K applying a constant cooling rate of 1 K/ns. Then, all three systems were subjected to drawing MD simulations in the *Nė*_*xx*_*P*_*yy*_*P*_*zz*_*T* ensemble under constant strain rate equal to 10^7^ s^–1^, constant ambient pressure in the *y* and *z* directions and constant temperature *T* = 365 K. The total simulation time was 200 ns and the final stretch ratio was equal to 3. During this drawing stage, a semicrystalline morphology is developed. Then, the stretched semicrystalline PE samples were submitted to MD simulations in the ensemble *NL*_*xx*_*P*_*yy*_*P*_*zz*_*T* at the same constant temperature of *T* = 365 K, constant ambient pressure at *y* and *z* directions, and keeping the stretched direction *L*_*x*_ constant. During the latter stage (annealing stage), the already formed crystals continue to grow, and the degree of crystallinity of the samples increases until it reaches almost a plateau value when we terminate our simulations at approximately 10 μs. Finally, the three semicrystalline stretched samples were brought to the desired temperature of *T* = 298 K by applying a constant cooling rate of 1 K/ns and let them relax via MD simulations in the ensemble *NL̇*_*xx*_*P*_*yy*_*P*_*zz*_*T* at a constant temperature of *T* = 298 K, under constant ambient pressure at *y* and *z* directions and keeping the stretched direction *L*_*x*_ constant. Once again, the evolution of their degree of crystallinity, χ_c_, was monitored and the simulations continued to run until χ_c_ reached a clear plateau value.

### 2.6 Test Particle Insertion Method

Widom test particle insertion (TPI) is a well-known method for predicting the thermodynamics of solvation. Here, the implementation of the method in molecular dynamics simulations is discussed. To start with, the solvation equilibrium at low pressures is dictated by Henry’s solubility law^24^
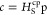
2where *c* is the concentration of the gas dissolved in the polymer and *p* is the pressure of the gas above the polymeric membrane. *H*_S_^cp^ is the Henry’s law solubility constant. According to IUPAC notation,^25^ the superscript denotes the variables that are used to describe the relation between the polymer and the gas phase—in this case, cp stands for concentration and pressure. The units of *H*_S_^cp^ in SI are . The Henry’s law solubility constant can be also expressed as *H*_S_^cc^, which is dimensionless, or as *H*_S_^xp^, where *x* stands for molar mixing fraction, which is the variant often reported experimentally and its units are . In ref,^25^ the relations between the different variants are reported explicitly. Here, we include the relation between *H*_S_^xp^ and *H*_S_^cc^ given by
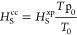
3where the standard pressure and temperature (STP) of *T*_0_ = 273.15 K and p_0_ = 101,300 Pa (atmospheric pressure) are used and *T* is the solvation temperature. In the present study, the dimensionless variant *H*_S_^cc^ will be used and we will sometimes simply refer to it as solubility.

The TPI allows us to estimate *H*_S_^cc^ by the means of molecular dynamics. This can be done by calculating the potential energy change Δ*E* upon random insertion of a single penetrant particle in the host polymer and then using the relation^11^
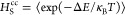
4where κ_B_ is the Boltzmann constant and ⟨···⟩ denotes averaging over multiple insertions and over all configurations of the matrix. Finally, the excess Gibbs free energy of the solvation process Δ*G*_ex_ follows by
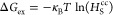
5

A molecular dynamics simulation of a pure polymer phase in an *NPT* ensemble provides polymer configurations upon which to perform TPI upon. In order to estimate the solubility via MD trajectories, we have developed an in-house software package capable of reading popular trajectory formats from programs such as GROMACS and LAMMPS. The software allows TPI to be performed across multiple frames within a single trajectory and supports any triclinic simulation box with periodic boundary conditions. Additionally, the software is optimized through support for parallel insertions.

The potential energy change Δ*E* upon insertion of single penetrant particle in the host polymer is dependent only on the intermolecular penetrant–polymer interactions , given that the (intramolecular and intermolecular) polymer–polymer interactions  are unaffected by the insertion. The penetrant–polymer interaction potential  generally consists of van der Waals interactions and Coulomb interactions. Since the deployed force field for the polymer does not prescribe any Coulombic interactions, the penetrant–polymer interaction potential  is exclusively dependent on the van der Waals interaction, assuming that we have infinite dilution. The van der Waals interactions are modeled by the well-known Lennard-Jones potential, such that , where *r* is the distance between two interacting particles *i* and *j*, σ_*ij*_ is the distance at which the particle–particle potential energy is zero, and ϵ_*ij*_ is the depth of the potential well. In numerical studies, it is customary to limit the range of interaction to a distance *R*_cut_ which was set equal to 1.2 nm to decrease the computational load. The Lennard-Jones potential therefore requires a correction term  that accounts for the interactions outside the cutoff radius, such that^11^
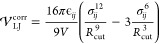
6where *V* is the volume of the simulation box. It should be noted that beyond *R*_cut_, *g*(*r*) = 1.

### 2.7 Local Solubility Method

The Lennard-Jones interaction energy and thus the solubility for each insertion are highly dependent on the local structure of the polymer. For this reason, the correlation of the solubility with the local polymer segment orientation is of interest. To quantify this correlation, for each insertion, we calculate a local order parameter *P*_2,local_ around the insertion point defined as
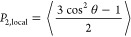
7where θ is the angle between two chord vectors connecting iPP backbone atoms that are six bonds apart and ⟨···⟩ denotes averaging over all the chord vectors within *r*_OP_ = 1.2 nm—the cutoff distance of Lennard-Jones interactions—from the insertion point. Then, to assess the correlation between the local solubility and local orientation, the estimated solubility for each insertion was averaged over bins of Δ*P*_2,local_ = 0.01.

## 3 Results

### 3.1 Solubility in Amorphous iPP and PE

In MD simulations, the challenge of equilibrating well-entangled polymers at temperatures close to the glass-transition temperature is a well-known issue. Properties such as density, thermal conductivity, and glass-transition temperature are highly dependent on the cooling rate.^26,27^ Especially, the cooling rate dependence of the glass-transition has been discussed in the past by many researchers.^28−30^ Typically, in the plot of the specific volume with respect to the temperature, there are two characteristic branches, the fluid and the solid branches, the intersection point of which can be defined as the glass-transition temperature. According to Buchholz et al.,^30^ the glass-transition temperature increases logarithmically with the cooling rate, with a relative increase of 10% within approximately 3 orders of magnitude. So, the divergence of the glass-transition temperature estimated in molecular simulations from the one estimated experimentally is expected, given that typical MD cooling rates are many orders of magnitude higher than the experimental ones. This effect can be attributed to the excess free volume, which can affect the solubility as well. Fewer studies exist on the effect of the cooling rate on the solubility of small gases in polymers. Pandiyan et al.^31^ showed that annealing–cooling cycles can lead to a decrease of CO_2_ solubility in polyimide samples. Recently, a quantification of this effect was reported by Volgin et al.,^32^ where the solubility of various gases in thermoplastic polyimide R-BAPB was reduced by a factor of 2, when the cooling rate was reduced by 4 orders of magnitude. In the same study, a method based on Struik’s theory^33^ was proposed for extrapolating the measured values to realistic cooling rates. In this study, such extrapolation is not applied, but it could be a possible direction for future study.

**Figure 1:**
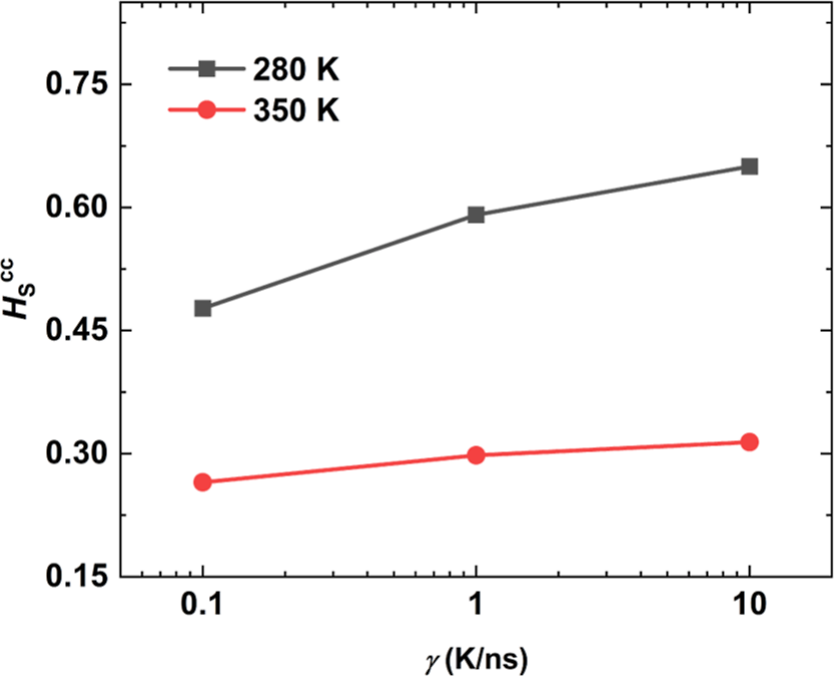
Dimensionless Henry’s law solubility constant *H*_S_^cc^ as a function of the cooling rate γ for iPP at a supercooled melt state and in a glassy state at 350 K (black squares) and 280 K (red circles), respectively. The sample was first equilibrated at 500 K and then cooled down to the respective temperature with varying cooling rates: 0.1, 1, and 10 K/ns.

To assess the influence of the cooling rate in this study, an equilibrated initial configuration of iPP at 500 K was cooled down to 350 and 280 K with cooling rates ranging from 0.1 to 10 K/ns. The computationally estimated glass-transition temperature for iPP using a cooling rate of 0.1 K/ns is equal to *T*_g_ = 305.6 K,^34^ while the experimental value is *T*_g_ = 272 K.^35^Figure 1 shows the dimensionless Henry’s law solubility constant *H*_S_^cc^ as a function of the cooling rate γ. The samples at 280 K depicted in black are below *T*_g_ = 305.6 K, meaning that they are in a glassy state and as the cooling rate γ decreases, *H*_S_^cc^ decreases as well. The samples at 350 K depicted in red are in a supercooled state and, similar to the previous samples, exhibit a positive dependence of *H*_S_^cc^ on the cooling rate γ; however, γ has a weaker effect.

**Figure 2:**
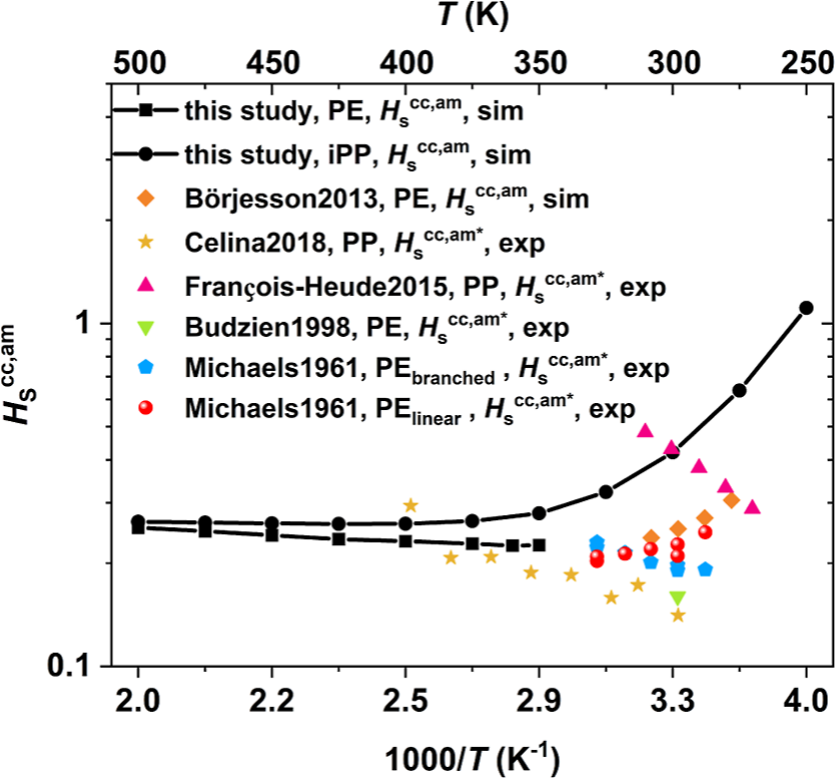
Temperature dependence of the oxygen solubility *H*_S_^cc,am^ in amorphous PE and iPP, depicted in black squares and black circles, respectively. The error bars extracted from three independent configurations for iPP and PE are negligible, so they are omitted. Comparison is made with other experimental and simulation results; (orange rhombus) Börjesson et al.;^37^ (yellow star) Celina and Quintana;^38^ (pink upper triangle) François-Heude et al.;^39^ (green reverse triangle) Budzien et al.;^40^ and (blue pentagon, branched PE) (red circle, linear PE) Michaels and Bixler^1^_._

A cooling rate of 0.1 K/ns yields the lowest solubility, but it is computationally costly to use such a cooling rate given that a cooling simulation from 500 to 250 K would require 2.5 μs. This leaves 1 K/ns as the cooling rate of choice for preparing the amorphous PE and iPP samples for solubility measurements in the present work. In total, 10^7^ insertions were performed—10^4^ insertions per frame for 1001 frames for each sample. In Figure 2, the Van ′t Hoff plot^12^ in a log scale of the dimensionless Henry’s law solubility constant ln(*H*_S_^cc,am^) of oxygen in amorphous PE (black circles) and iPP (black squares) samples is plotted versus the reciprocal temperature. In the same graph, oxygen solubilities collected for polypropylene and PE from the experimental and simulation results are plotted as well. *H*_S_^cc,am*^ means that the solubility has been measured in the semicrystalline state and then scaled to pure amorphous phase using the linear relation, *H*_S_^cc,am*^ = *H*_S_^cc,sm^/(1–*χ*_c_). *H*_S_^cc,am^ denotes solubility measured in an actual purely amorphous sample. It should be noted that all of the experimental solubilities are scaled due to the fact that the experimental PE and iPP samples crystallize very fast at supercooled temperatures.

The oxygen solubility *H*_S_^cc,am^ of the amorphous iPP samples is highly temperature-dependent. It can be seen that from 250 to 375 K, *H*_S_^cc,am^ decreases, while beyond 400 K, solubility starts to increase slightly. The partial heat of solvation Δ*H*_S_ can be extracted from the Van ’t Hoff plot^12^ using the expression
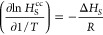
8where *T* is the temperature and *R* is the gas constant. Using eq 8, Δ*H*_S_ = −10.2 ± 0.8 kJ/mol within the range from 250 to 325 K and Δ*H*_S_^*^ = 0.24 ± 0.06kJ/mol within the range from 375 to 500 K. Between 250 and 325 K, Δ*H*_S_ < 0 indicates an exothermic process, meaning that the solvation is not favored by the increase in temperature, while between 375 and 500 K, Δ*H*_S_ > 0 indicates an endothermic process, meaning that the solvation is favored by the increase in temperature. This temperature dependence can be explained by equating Δ*H*_S_ = Δ*H*_R_ + Δ*H*_B_, where Δ*H*_B_ is the binding enthalpy of the polymer with the penetrant and Δ*H*_R_ is the reorganization enthalpy of polymer upon the solvation of the penetrant, or in other words, the enthalpy for creating a cavity, following the notation of van der Vegt et al.^41^ The binding enthalpy Δ*H*_B_ is always negative, while the reorganization enthalpy Δ*H*_R_ is always positive. So, the sign of the solvation enthalpy Δ*H*_S_ results from an interplay between these two contributions. At temperatures close to the glass-transition temperature, the cavities are nearly frozen, resulting in Δ*H*_R_ ≈ 0; thus, the negative term Δ*H*_B_ prevails. In a previous study of ours,^18^ the glass transition temperature for iPP was measured equal to *T*_g_ = 313.7K using a cooling rate of 1 K/ns. In the same study, a lower cooling rate of 0.1 K/ns resulted in *T*_g_ = 305.6K. This perfectly aligns with the temperature range where Δ*H*_R_ of iPP becomes negative. On the other hand, *H*_S_^cc,am^ measured for PE within the reported temperature range exhibits a monotonic trend, where *H*_S_^cc,am^ increases with temperature and Δ*H*_S_ = 1.1 ± 0.1 kJ/mol. Lower temperatures in the case of PE could not be studied because the samples crystallize immediately.

Celina and Quintana^38^ also studied the oxygen solubility in injection moldable PP grade film with a crystallinity measured by differential scanning calorimetry (DSC) approximately equal to 45% and density 0.89 g/cm^3^. The oxygen solubility measurements were performed in semicrystalline samples and scaled to amorphous samples *H*_S_^cc,am*^ using the linear relationship reported earlier. The present simulation results for oxygen solubility of iPP at 400 K (black circles, Figure 2) are in good agreement with experimental results obtained by Celina and Quintana.^38^ However, at lower temperatures, a larger deviation is observed and, additionally, a larger solvation enthalpy of Δ*H*_S_^*^ = 3 kJ/mol is reported by Celina and Quintana,^38^ compared to Δ*H*_S_^*^ = 0.24 ± 0.06 kJ/mol, estimated in the present study. In Ref (38) it is reported that crystallinity might slightly change upon heating, meaning that a possible partial melting of the crystalline phase upon heating can significantly affect the temperature dependence of the solubility. Experimental data for iPP from François-Heude et al.^39^ are also depicted in Figure 2. There is an overlap of the values at certain temperature; however, the solubility temperature dependence is inverse compared to the present study in the temperature range from 250 to 325 K and the reported enthalpy of solvation is Δ*H*_S_^*^ = 6.7 kJ/mol. At this point, it should be stressed that experimental results across different temperatures where the crystallinity does not change are of great significance in order to directly compare with the simulation results. Michaels and Bixler^1^ touch upon the effect of melting on the temperature dependence of solubility. Specifically, *H*_S_^cc,am^ reported by Michaels and Bixler for a linear and branched PE (red circles and blue pentagons, respectively, in Figure 2) exhibit two different trends. *H*_S_^cc,am*^ of linear PE decreases with temperature, yielding an enthalpy of solvation equal to Δ*H*_S_^*^ = −1.67 kJ/mol, while the opposite trend is observed for *H*_S_^cc,am*^ of branched PE, with Δ*H*_S_^*^ = 2.51 kJ/mol. In the same study, it is highlighted that crystallinity remains constant for the linear PE across the studied temperature range, while melting is observed for the branched PE. Results for linear PE^1^ are also in good agreement with simulation results reported in Ref (37) for slightly entangled PE of 200 carbon atoms per chain (orange rhombus in Figure 2). *H*_S_^cc,am^ reported in the latter two cases can be seen as an extension of the results for PE presented in this study to a lower temperature range. In this case, a hypothetical minimum, can be spotted in the range of 325 –300 K—one estimate for the glass-transition temperature for PE is equal to 200 K.^42^ Further studies on the estimation of a possible transition point of *H*_S_^cc,am^ for PE would give us valuable insights. Finally, Budzien et al.^40^ predicted *H*_S_^cc,am^ = 0.15977 in completely amorphous PE at room temperature by extrapolating alkane behavior to the long chain limit in a Flory–Huggins context.

### 3.2 Oxygen Solubility of Semicrystalline PE and iPP Samples

In order to study the solubility of oxygen in semicrystalline iPP and PE samples, three semicrystalline iPP samples and six PE samples were prepared at room temperature with different crystallinity values. The iPP samples originated from three initial configurations which were crystallized under flow at 410 K and then cooled to room temperature, while half of the PE samples were also crystallized under flow at 365 K and the rest without deformation under quiescent conditions at 340 K.

In order to extract the kinetics of crystallization for all cases, the mean first passage time (MFPT) method was used. The main equation of this method is given below by Ref (43)
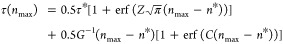
9where τ(*n*_max_) is defined as the time at which a crystalline cluster of size *n*_max_ appears for the first time in the bulk, τ* is the nucleation time, n* is the critical nucleus size, *G* is the growth rate, *Z* is the so-called Zeldovich factor, which is the probability that a nucleus at the top of the barrier will grow instead of dissolving, and *C* is a constant chosen to be a large positive value.

By fitting eq 9 to the plots of τ(*n*_max_) vs *n*_max_ the kinetic parameters are extracted for configurations prepared by (a) flow-induced crystallization (FIC) of iPP at 410 K, (b) FIC of PE at 365 K, and (c) quiescent crystallization (QC) of PE at 340 K. The values averaged over the three independent configurations are presented in Table 1.

**Table 1:** Average Values of the Kinetic Parameters Obtained Using the MFPT Method for the iPP and PE

	τ* (ns)	*G* (atoms/ns)	n* (atoms)	*Z*
iPP (FIC-410 K)	95 ± 17	7 ± 1	104 ± 25	0.010 ± 0.004
PE (FIC-365 K)	135 ± 6	247 ± 6	79 ± 9	0.004 ± 0.001
PE (QC-340 K)	187 ± 43	94 ± 12	80 ± 9	0.003 ± 0.001

It can be seen in Table 1 that τ* = 95 ± 17 ns for iPP under FIC, smaller compared to τ* = 135 ± 6 ns for PE under FIC. It should be noted that the undercooling Δ*T* = 50 K for iPP (*T*_m_ = 460 K^21^), while Δ*T* = 31 K for PE (*T*_m_ = 396.4 K^36^). Additionally, the acceleration of the nucleation due to stretching is evident by looking at τ* = 135 ± 6 ns for PE under FIC at 365 K compared to τ* = 187 ± 43 ns for PE under QC at 340 K. In particular, although the PE samples under quiescent conditions correspond to lower temperatures that favor nucleation, the flow field imposed on the PE samples under FIC has a drastic effect, accelerating nucleation and decreasing the corresponding induction time. An additional interesting observation is that the growth rate *G* = 247 ± 6 atoms/ns for PE under FIC, indicating that the growth of the crystals takes place significantly faster in the case of PE compared to *G* = 7 ± 1 atoms/ns for iPP. On top of that, stretching facilitates crystal growth, too, taking into account that *G* = 94 ± 1 atoms/ns for PE under QC is smaller compared to FIC. Finally, the critical nucleus size does not exhibit large differences between PE under FIC and QC but it is generally higher in iPP samples.

**Figure 3:**
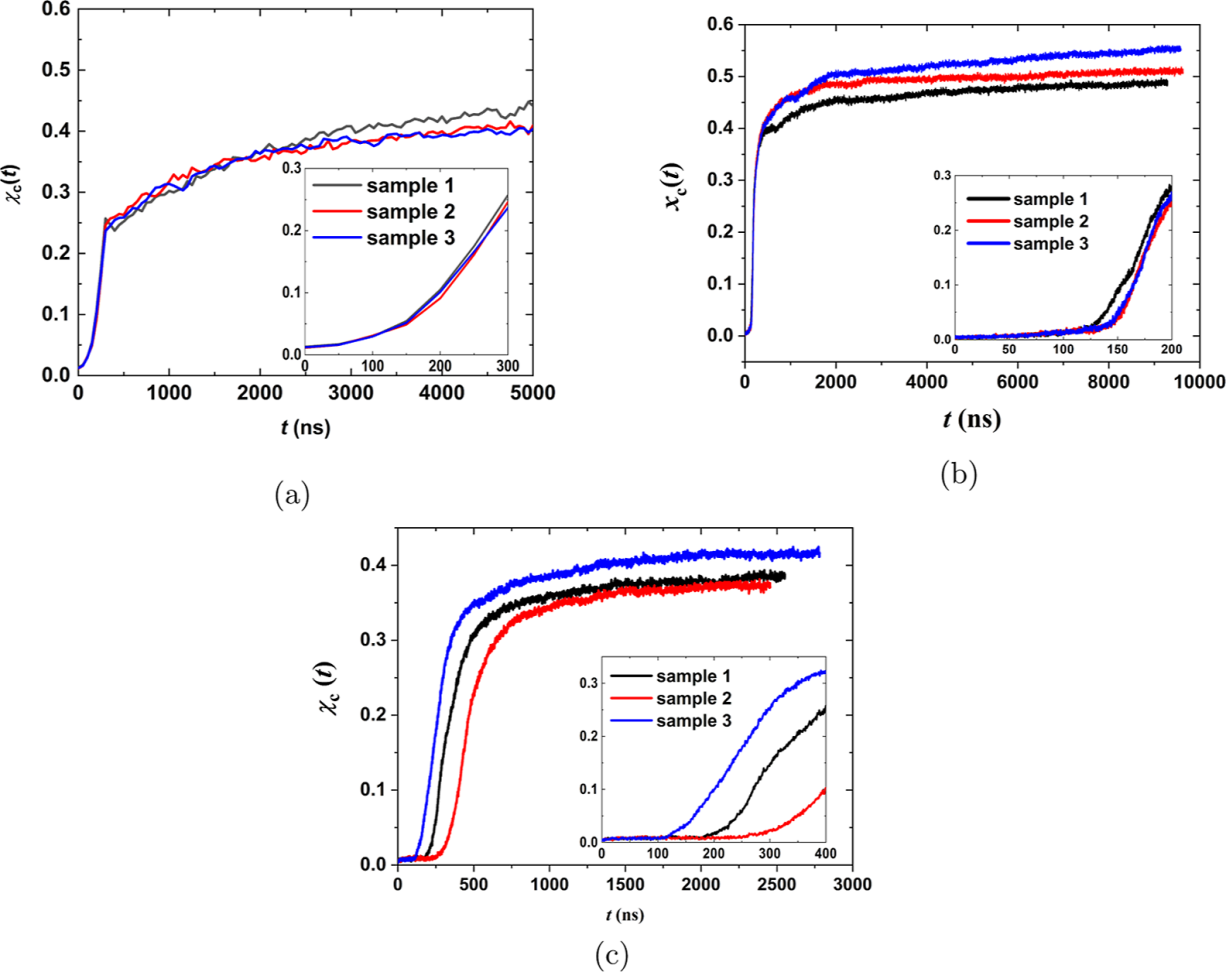
Time evolution of crystallinity *χ*_c_ for (a) three independent iPP supercooled samples at 410 K under FIC, (b) three independent PE supercooled samples at 365 K under FIC, and (c) three independent PE supercooled samples at 340 K under QC. The inset plots of (a,b) present the crystallinity evolution during the stretching stage, which lasted 300 and 200 ns for iPP and PE, respectively. The inset plot of (c) depicts χ_c_ vs time *t* during the stage where nucleation takes place.

Then, the evolution of crystallinity is monitored during the nucleation and growth stage. Specifically, in Figure 3a, the time dependence of the crystallinity of iPP for three independent configurations is depicted during the stretching and the annealing stage at 410 K. The crystallinity was measured using Yamamoto’s method.^44^ It can be seen that during the early stages of stretching (inset plot), where nucleation takes place, the crystallinity increases slowly. After 100 ns, it starts increasing rapidly, signifying crystal growth, and reaches values of up to 0.25. At *t* = 300 ns, the elongation is stopped, and annealing is performed at the same temperature, keeping the box length in the elongational direction constant. The crystallinity continuously increases, and even after 5000 ns of annealing, no plateau value has been reached. It should be noted that the initial structure in the early stages of FIC is not lamellar. A comprehensive study of the structure and the effect of annealing is presented in the following sections.

In Figure 3b, the crystallinity plotted against time is depicted for three independent samples for PE under FIC. In this case, the crystallinity was measured based on a mesh shell method suggested by Yamamoto.^45^ Comparing to iPP, the overall crystallinity develops much faster. The nucleation takes place around 100 ns (inset plot), as was shown by the MFPT, when χ_c_ starts to develop significantly. At 200 ns, when the stretching stage is completed and the stretching ratio becomes equal to 3, χ_c_ ranges between 0.25 and 0.3. The annealing stage is followed at the same temperature, equal to 365 K, by keeping the tensile direction constant, during which χ_c_ starts to gradually reach a plateau value. This is evident after 2000 ns. It can be said, however, that even after 10,000 ns, there is a slight tendency upward, especially for the blue curve. Due to limitations, in computational resources, the annealing simulations were terminated at approximately 10,000 ns. In the case of iPP, reaching a plateau value was even more challenging—a clear plateau could not be observed even after an annealing simulation that lasted in total 10,000 ns. So, in the present study, only annealing simulations of iPP up to 5000 ns are presented.

Additionally, in Figure 3c, *χ*_c_ vs time *t* is also presented for PE under QC. Data extracted from quiescent simulations were included in order to reach lower crystallinity values than the ones measured in the semicrystalline samples prepared under FIC and to enrich the graph of the solubility vs crystallinity, as will be shown later. Under quiescent conditions, nucleation and growth take a longer time. Crystallinity increases at 100 ns for the third sample (blue curve), while for the second sample (red curve), there is no significant change even after 200 ns (inset plot). The plateau value starts to clear up after 1500 ns. The end crystallinity values, which are around 0.4, are lower than the ones observed under FIC.

**Figure 4:**
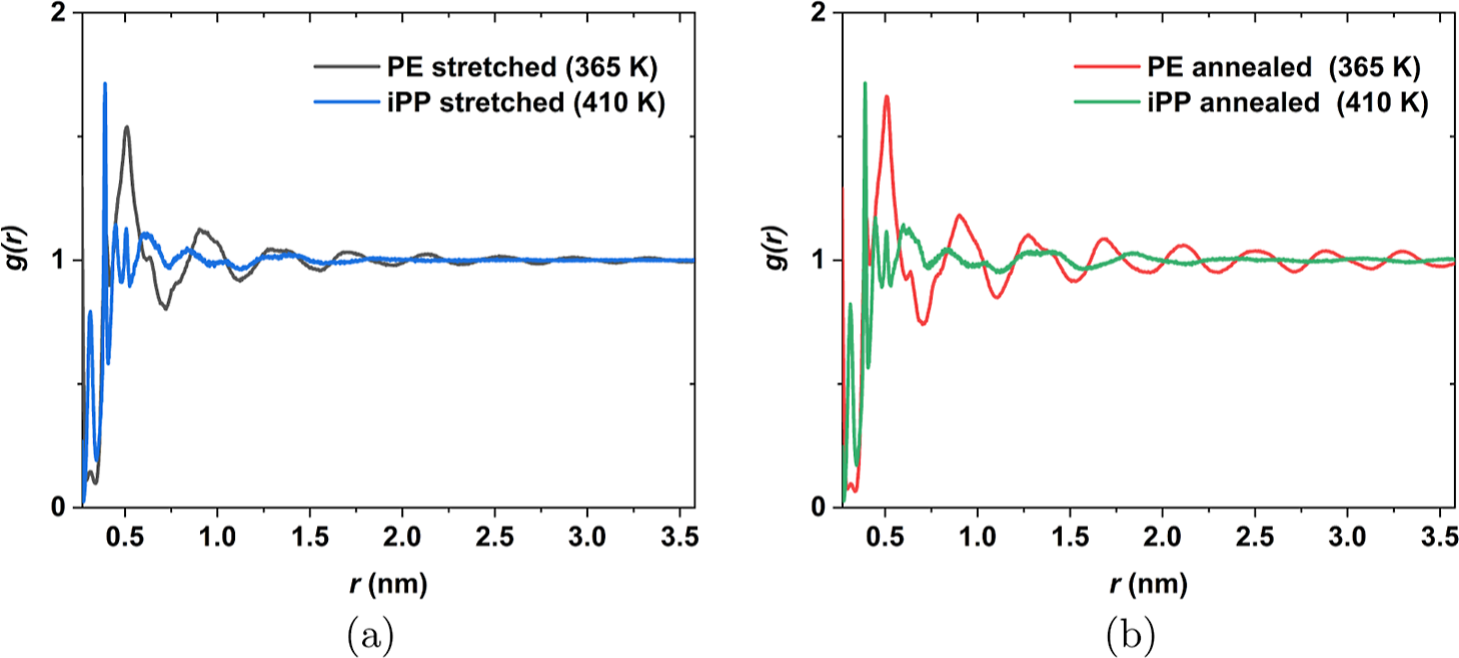
RDF *g*(*r*) (a) after stretching of iPP and PE at 410 and 365 K, respectively, and (b) after annealing of iPP and PE for 5 and 10 μs, respectively.

Next, the focus is placed on the structure that develops upon FIC for iPP and PE and on a comparison between these two systems. In Figure 4a, the radial distribution function (RDF) *g*(*r*) of an iPP (blue line) and PE (black line) configuration is depicted after the end of the stretching stage. The stretching stage for iPP lasted for 300 ns at 410 K, while that for PE lasted for 200 ns at 365 K. Looking at the repetitive peaks that persist up to 3.5 nm along the black curve, it is apparent for PE that a crystalline morphology has started to develop and crystals at least as large as 7 nm are present. In the case of iPP, despite nucleation, the repetitive peaks, which are indicative of a crystal morphology spanning large spatial scales, are not present. A well-developed crystal morphology for iPP is obtained later, after the annealing stage, as can be seen in Figure 4b, where *g*(*r*) is depicted in green for a configuration of iPP at the end of the annealing stage after 5000 ns. In the case of PE, *g*(*r*) depicted in red shows that the peaks are more distinct after the annealing stage of approximately 10,000 ns, and a well-developed crystal network has been formed.

**Figure 5:**
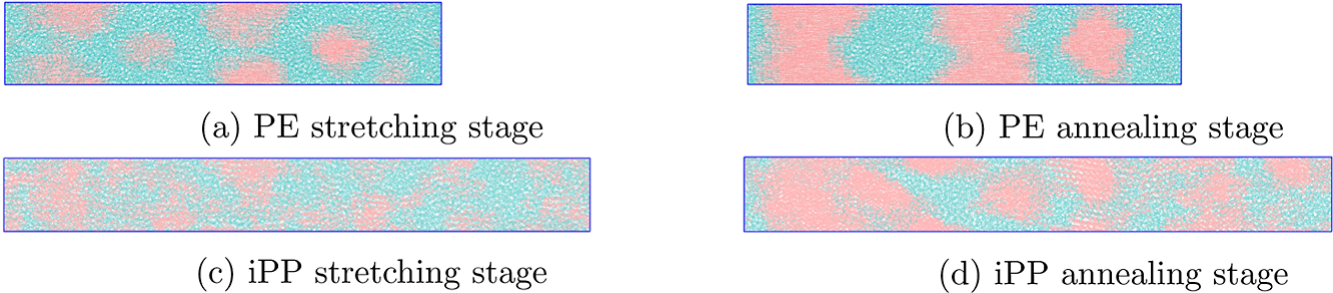
Snapshots at the end of (a) stretching of PE at 365 K, (b) annealing of PE at 365 K, (c) stretching of iPP at 410 K, and (d) annealing of iPP at 410 K. Crystalline (amorphous) regions defined according to the orientation criterion of Yamamoto^44,45^ are shown in pink (cyan) color. The box length across the tensile direction of iPP is approximately equal to 60 nm, while for PE, that is equal to 45 nm.

In order to acquire more details about how the actual structure looks, two snapshots of PE are presented in Figure 5a,b, after the stretching and annealing stage, respectively. The lamellar morphology is already present in PE even after the stretching stage. With annealing, the structure is improved and the lamellar length increases significantly, resulting in distinct alternating crystal and amorphous regions. Similar to PE, the snapshots of iPP are also shown in Figure 5c,d, at the end of the stretching and annealing stage, respectively. For iPP at the end of the stretching stage, the lamellar morphology is not prevalent; mostly isolated stable crystals exist. At the end of the annealing stage of iPP, the lamellae have started to form and the structure is reminiscent of that of PE in Figure 5a.

At this point, one thing to note is that we quite often refer to lamellar morphology. Experimentally lamellar structure is perceived as chains packed in a well-ordered fashion with the chains folding back and forth. Quite often, a distinction is made between lamellar structure and shish structure in a way that the shish structure is perceived as a fibrillar structure phase comprising extended chains with alternating crystal and amorphous parts. In the context of this study, we regarded the lamellar crystals as part of the shish structure, so we make no distinction.

**Figure 6:**
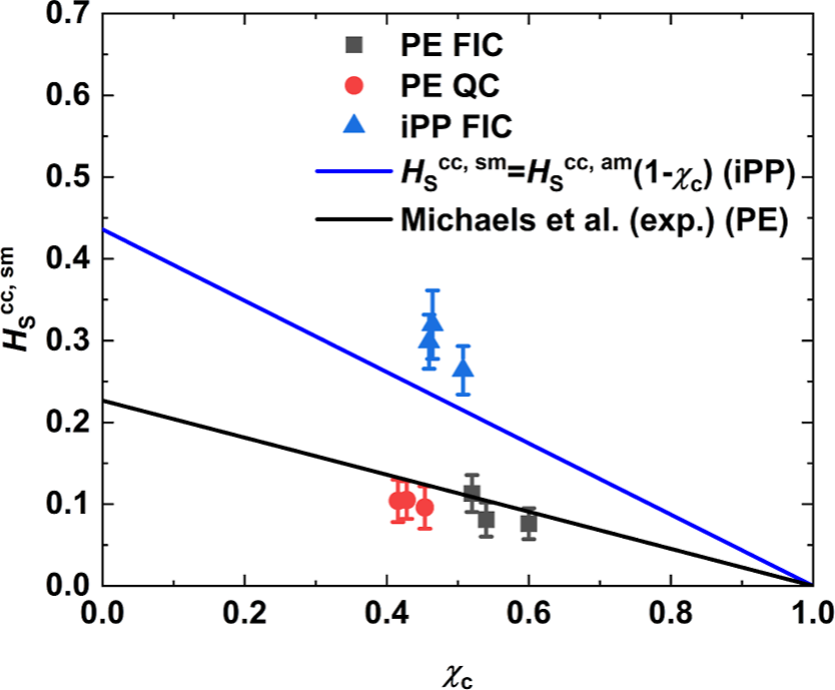
Henry’s law solubility *H*_S_^cc,sm^ plotted against crystallinity χ_c_ for semicrystalline polymer configurations at 298 K. The configurations refer to (a) iPP prepared under FIC at 410 K (blue triangles), (b) PE prepared under FIC at 365 K (black squares), and (c) PE prepared under QC at 340 K (red circles). The error bars are included as well. The black line was drawn based on the experimental results presented by Michaels and Bixler^1^ for linear PE. The blue line corresponds to the prediction of the linear relationship (eq 1) based on the solubility *H*_S_^cc,am^ measured in amorphous iPP at 300 K.

Lastly, the Henry’s law oxygen solubility *H*_S_^cc,sm^ was measured for semicrystalline samples of iPP and PE at 298 K and plotted in Figure 6. Samples prepared under FIC of PE and iPP are denoted in black squares and blue triangles, respectively. PE samples prepared under QC are depicted as red circles. For each case, three independent configurations were sampled and, for measuring *H*_S_^cc,sm^, the last 50 ns of the NPT simulation at 298 K were used. The error bars are included as well. The blue line corresponds to the prediction of the linear relationship (eq 1) based on the solubility *H*_S_^cc,am^ measured in amorphous iPP at 300 K. In the case of PE, *H*_S_^cc,am^ measurements at ambient temperature in fully amorphous samples were not feasible due to early nucleation as mentioned previously. For that reason, experimental results obtained by Michaels and Bixler^1^ were used in combination with the linear relationship in order to draw the black line. The latter is in good agreement with *H*_S_^cc,sm^ measured for semicrystalline PE samples under both QC and FIC. On the contrary, *H*_S_^cc,sm^ of semicrystalline iPP slightly diverges from the prediction of the linear relationship (blue line). It was shown previously that the development of the crystal morphology actually takes place on different time scales in iPP and PE. Thus, the divergence in iPP is attributed to the lack of structure. The linear relationship assumes that the crystal phase is not permeable and does not affect the amorphous phase. In order to shed light on the validity of the linear relationship for the simulated iPP, an in depth study of the effect of annealing on its structure and how this is related to solubility follows.

### 3.3 Effect of Annealing on the Structure of Semicrystalline iPP

In order to study the effect of annealing on the structure of iPP, from each annealing simulation at 410 K, six configurations were drawn from the annealing trajectories between 0 and 5000 ns, with steps of 1000 ns. Each configuration was then cooled down to 298 K and simulated in a NL_*xx*_*P*_*yy*_*P*_*zz*_*T* ensemble at the same temperature for 100 ns. The crystallinity values, averaged over the last 50 ns, are presented in Table 2. Notably, the crystallinity during this stage remains relatively constant across all of the samples. Samples without annealing at 410 K exhibit a crystallinity of 0.33–0.34. In contrast, samples annealed for 5000 ns can reach crystallinity values close to 0.5, which are considered quite high by simulation standards.

**Table 2:** Crystallinity Values for Three iPP Samples at 298 K, Annealed at 410 K for Different Annealing Times^a^

*t*_anneal_ (ns)	sample 1	sample 2	sample 3
0	0.34	0.33	0.33
1000	0.40	0.40	0.40
2000	0.45	0.43	0.43
3000	0.47	0.45	0.45
4000	0.49	0.46	0.46
5000	0.51	0.46	0.46

a Crystallinity is averaged out of the last 50 ns of an *NPT* ensemble simulation at 298 k.

**Figure 7:**
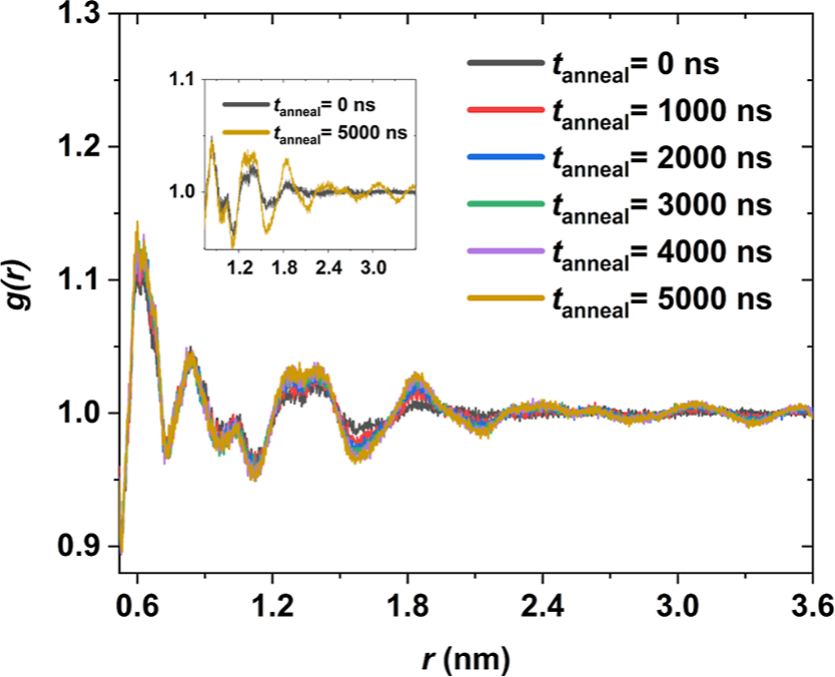
RDF *g*(*r*) of five iPP samples at 298 K. Each sample was annealed at 410 K for a different annealing time and then cooled down to 298 K with a cooling rate of 1 K/ns.

To delve more deeply into the structural effects of annealing, the RDF is plotted for sample 1 at six different annealing times in Figure 7. Upon examination, peaks of the RDF start to develop at longer distances, signifying lamellar development. The plot is truncated at 3.6 nm due to the box side length restriction. Up to 3.6 nm at 5000 ns, the RDF did not decrease to one, indicating that the lamellar length exceeds 7.2 nm. The characteristic peaks for all the annealing times lie at 0.6, 0.9, 1.3, 1.4, and 1.8 nm, which mainly related to the packing of the chains in the crystal. For annealing times equal to or longer than 2000 ns, the characteristic peaks are observed at 2.6, 3.1, and 3.6 nm.

**Figure 8:**
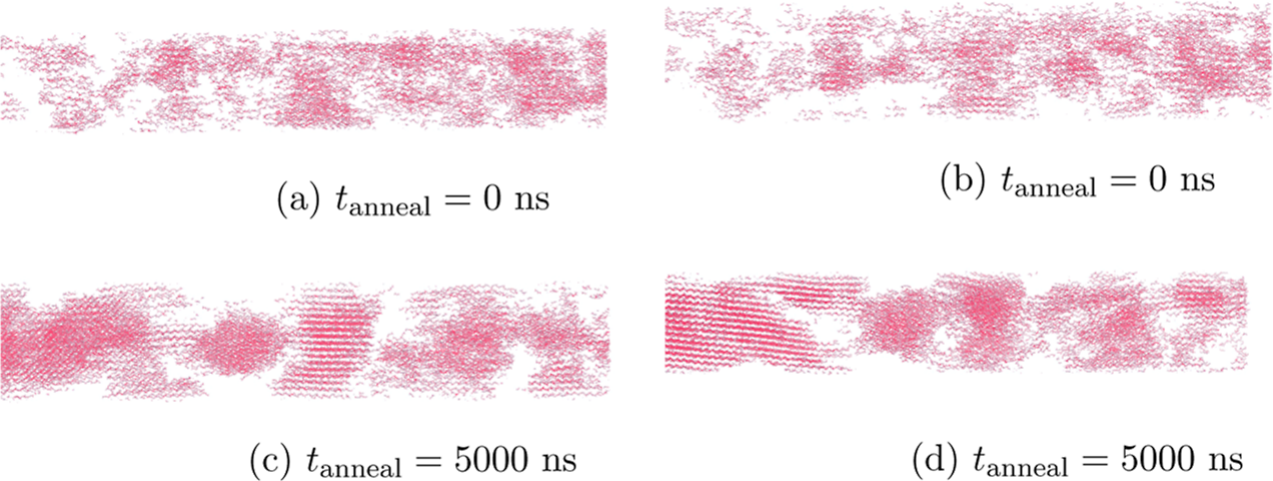
Snapshots of a sample at the end of the annealing simulation at 298 K. (a,b) Same configuration which has not been annealed at 410 K, viewed from a different perspective. (c,d) Same sample which has been annealed at 410 K for 5000 ns.

Figure 8a,b depicts snapshots of a sample, which was not previously annealed at 410 K, at the end of the *NPT* simulation at 298 K. It can be seen that some lamellar crystals exist, but they are small in size, as was also shown through the RDF. In the present study, the lamellar crystals are perceived as ordered domains where a clear alignment of long chain segments can be distinguished. Additionally, small isolated crystalline chain segments are observed. Figure 8c,d depicts snapshots at the end of the *NPT* simulation at 298 K from different perspectives for the same sample, which here has been previously annealed at 410 K for 5000 ns. In this case, a clear lamellar structure can be distinguished. It should be noted that the length of the simulation box along the tensile direction is 60 nm. Large clusters spanning at least 1/3 of the simulation box can be distinguished. As far as the structure formation of the shish morphology is concerned, Balzano et al.^46^ pose the questions of whether (1) stable nuclei are formed first and then shish is built up, or (2) shish is formed first and then stable nuclei develop internally. We argue that the former is valid if we envision that shish morphology actually comprises lamellar crystals in close proximity to each other, which are aligned along the tensile direction (Figure 8c,d). In the study presented here, we showed that lamellar formation takes place far beyond nucleation.

**Figure 9:**
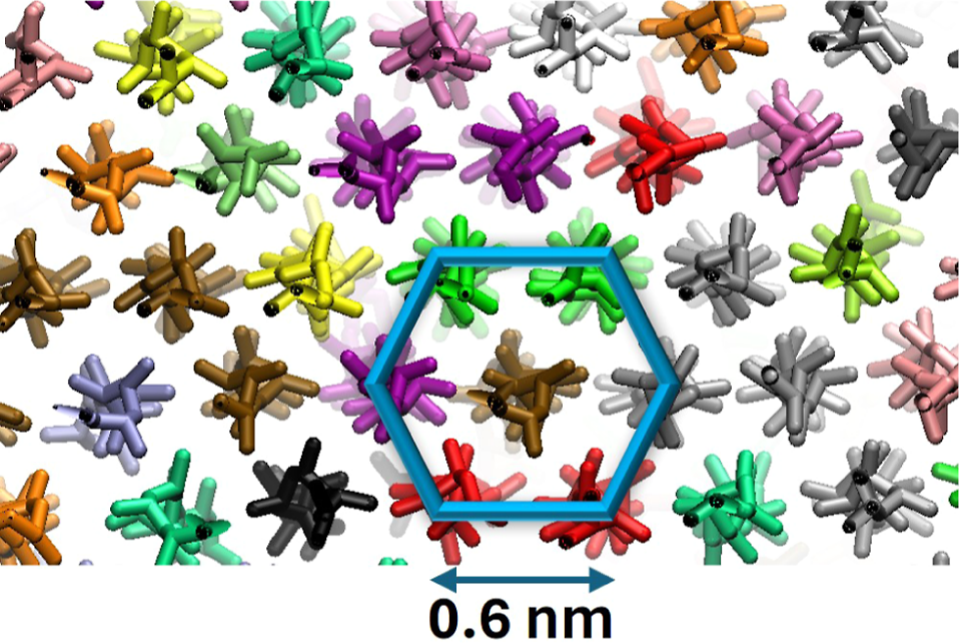
Hexagonal crystal phase of iPP at 298 K. Helical segments of different chains are depicted in different colors.

Additionally, the crystal phase formed upon creation of the lamellar morphology is presented in Figure 9. The snapshot was taken from the same sample discussed in Figure 8 at 298 K after a simulation of 100 ns. The system had been previously annealed at 410 K for 5000 ns. A hexagonal crystal phase can be seen with an edge length equal to 0.6 nm, coinciding with the peak at 0.6 nm of the RDF in Figure 7. The crystal phase of iPP obtained in the present study is similar to the crystal phase reported by another computational study.^47^ The distance of 0.6 nm corresponds to the intermolecular distance of the helical segments. The more stable α phase of iPP was not observed in the current simulations. It is hypothesized that first a hexagonal smectic phase is formed, and then, the crystal transits to a more stable phase. It is expected that the α phase requires 40–100 μs to form.^47^ The transition observed in this study can be also related to what Liu et al.^48^ observed experimentally and described as a transition from an “oriented shish precursor” to a “hexagonal shish crystal”.

### 3.4 Effect of Annealing on the Oxygen Solubility in Semicrystalline iPP

**Figure 10:**
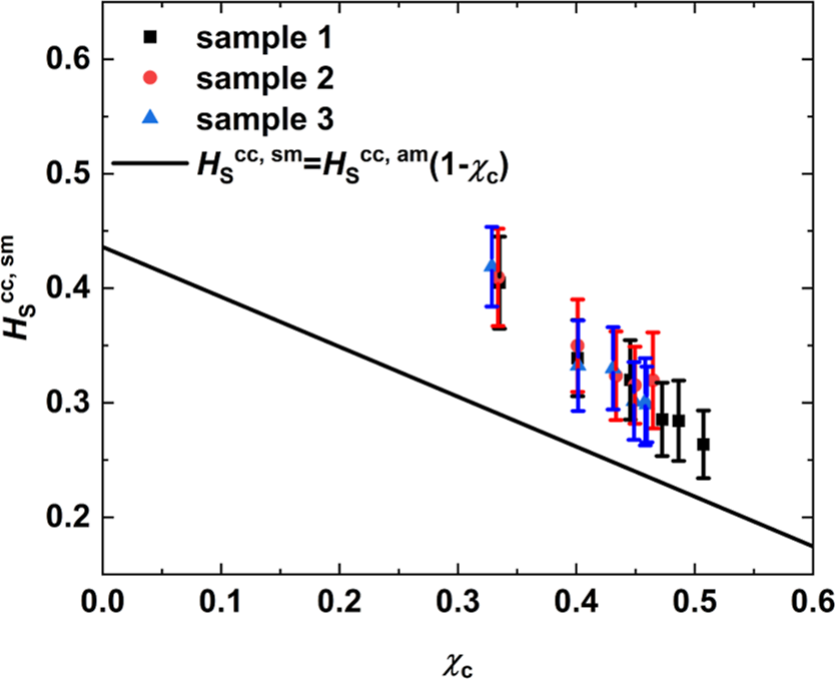
Henry’s law solubility *H*_S_^cc,sm^ of oxygen in iPP plotted against crystallinity χ_c_. The solubility measurements were performed on iPP configurations at 298 K in the course of an *NPT* simulation that lasted 100 ns. The configurations originated from three samples that were annealed for different times ranging from 0 to 5000 ns with step equal to 1000 ns at 410 K. Results from the three samples are shown separately as black squares, red circles, and blue triangles. Results for the configurations that were annealed for 5000 ns are the same with the ones depicted in Figure 6. The straight line corresponds to the prediction of the linear relationship (eq 1) based on the solubility *H*_S_^cc,am^ measured in amorphous iPP at 300 K.

The scaling relation from eq 1, formulated by Michaels and Bixler,^1^ assumes an impenetrable crystalline phase that does not impose any constraints on the amorphous phase. To assess the validity of these assumptions, the dimensionless Henry’s law solubility constant *H*_S_^cc,sm^ has been measured directly in the semicrystalline iPP samples. In the latest step of the preparation procedure, an *NPT* simulation of 100 ns was performed at 298 K. In each sample, 10^7^ insertions in total were performed in 1001 frames (last 50 ns). Figure 10 displays the value of the dimensionless Henry’s law solubility coefficient *H*_S_^cc,sm^ in the semicrystalline configurations as a function of the crystallinity. The black line is the theoretical behavior of the solubility as a function of the crystallinity according to the linear relationship and based upon the dimensionless Henry’s law solubility constant *H*_S_^cc,am^ found for the fully amorphous iPP samples at 300 K.

The discrepancy between the actual measurements and the theoretical prediction can most likely be attributed to the fact that either oxygen penetrates the crystalline phase or the amorphous phase is constrained by the crystalline phase. Additionally, it can be seen that the discrepancy decreases with increasing crystallinity. The configurations that have been annealed for 5 μs and possess the highest crystallinity exhibit a well-developed lamellar structure compared to the configurations that have not been annealed.

**Figure 11:**
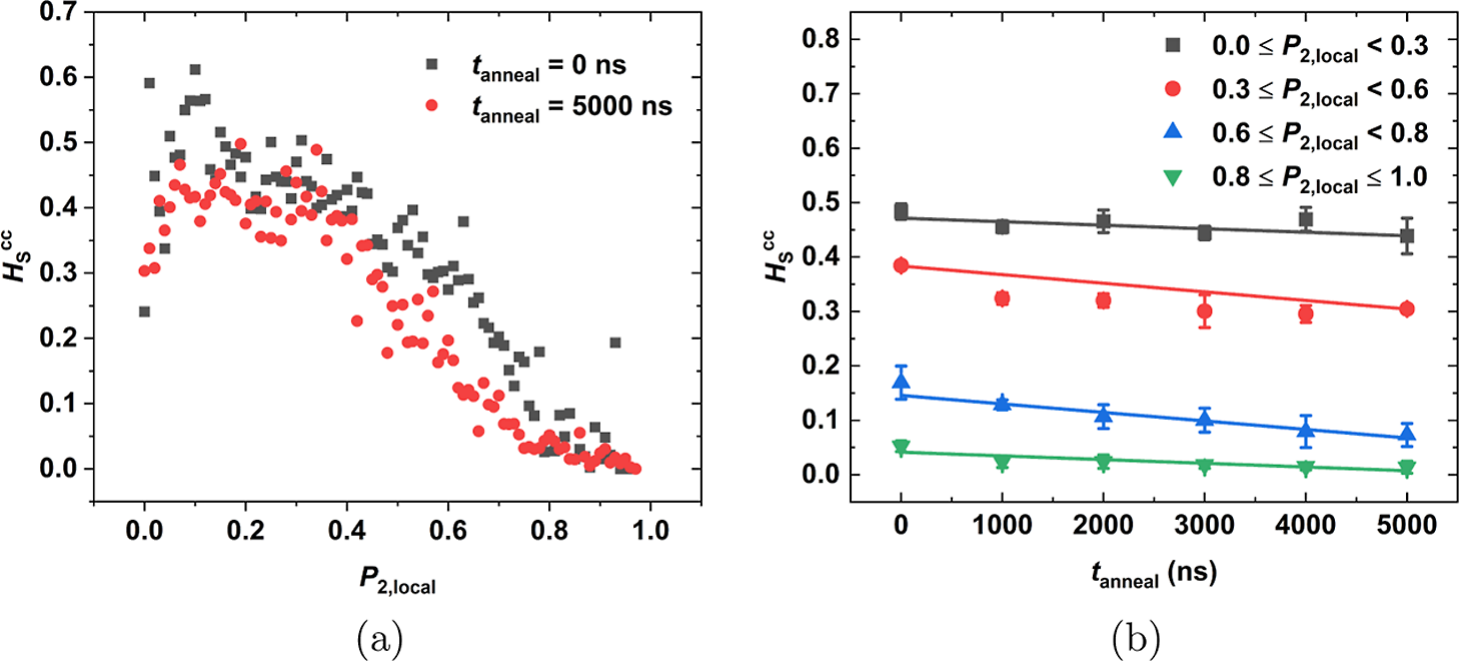
(a) Average value of *H*_S_^cc^ is plotted vs *P*_2,local_ for a sample at 298 K, which has been annealed for 0 and 5000 ns. (b) Average value of *H*_S_^cc^, categorized according to the local order parameter *P*_2,local_, is plotted against the annealing time at 410 K.

To measure the effect of the local morphology on the solubility, we examined the same samples by measuring the local order parameter *P*_2,local_ (Section 2.7). The goal is to correlate solubility with the local structure. The first step is to decide which values of *P*_2,local_ correspond to a crystalline structure and which to an amorphous one. Following Ref (49)*P*_2,local_ = 0.6 is set as the threshold value above which the structure is considered crystalline. Thereafter, for each oxygen particle insertion, a value for *H*_S_^cc^ was obtained and this was correlated to a value of *P*_2,local_ measured in the iPP matrix within a spherical shell of radius *r*_OP_ = 1.2 nm centered at the insertion point. Then, *H*_S_^cc^ was grouped in bins of Δ*P*_2,local_ = 0.01 and averaged out. In Figure 11a, the average value of *H*_S_^cc^ is plotted vs *P*_2,local_ for a sample at 298 K, which has been annealed for 0 and 5000 ns. Overall, *H*_S_^cc^ decreases with ordering, especially beyond *P*_2,local_ = 0.3, for both annealing times. In the range of *P*_2,local_ from 0.6 to 0.8, the sample that has been annealed for 5000 ns (red circles) exhibits a lower solubility than the same sample with no annealing (black squares). For *P*_2,local_ > 0.8, *H*_S_^cc^ is below 0.1. Some points in Figure 11a that deviate from the general trend should be attributed to the statistics, as this method requires a significant number of insertions in order to provide reliable results.

Lastly, the solubility is further distinguished in four categories according to the local order parameter prevailing in the matrix in the vicinity of the insertion point and plotted against the annealing time at 410 K in Figure 11b. The crystalline phase with *P*_2,local_ > 0.6 according to Ref (49) and the amorphous phase with *P*_2,local_ < 0.6 were both further subdivided into low order and high order environments. Thus, four environments were defined

- amorphous phase of low order (ALO) (0 ≤ *P*_2,local_ < 0.3)
- amorphous phase of high order (AHO) (0.3 ≤ *P*_2,local_ < 0.6)
- crystalline phase of low order (CLO) (0.6 ≤ *P*_2,local_ < 0.8)
- crystalline phase of high order (CHO) (0.8 ≤ *P*_2,local_ < 1)

The ALO and the CHO exhibit a lower dependency on the annealing time. The average value of *H*_S_^cc^ for ALO is 0.45, which is in good agreement with the solubility value measured in the purely amorphous phase. In contrast, the average value of *H*_S_^cc^ for CHO is 0.02, indicating that oxygen absorption is very low. The AHO and the CLO show a higher dependency on the annealing time *t*_anneal_, illustrating that annealing does affect the solubility of these environments.

One important point to highlight is that the distinction between the crystalline and amorphous phases is somewhat arbitrary. It is uncertain what the exact limits of these two phases are, and this ambiguity extends to experimental measurements. Experimentally, crystallinity is measured either by density measurements,^50^ which assign a certain density to each phase, or based on enthalpy by DSC.^51^ In both cases, assumptions of “pure domains” are made.

However, if we consider that for *P*_2,local_ > 0.6 the phase is crystalline, then it appears that part of the crystalline phase (CLO) is penetrable by oxygen without annealing, but after annealing, the crystal structure improves, admitting lower oxygen solubility. On the other hand, the AHO phase is also affected by annealing, contrary to our initial assumption. The AHO and CLO phases might be connected to the interlamellar phase observed experimentally. Further study of the spatial correlation between these phases would shed more light on this topic.

## 4 Conclusions and Outlook

This study focuses on the temperature dependence of oxygen solubility in amorphous iPP and PE samples by means of molecular dynamics and TPI methods. Measurements were also performed on semicrystalline PE and iPP, and the effect of annealing on the morphology of iPP and, thus, on the solubility was highlighted.

Initially, the effect of the cooling rate was studied and found to significantly impact the solubility in iPP significantly. Lower cooling rates, such as 0.1 K/ns, yield a lower *T*_g_ and reduce solubility, aligning more closely with experimental values, but are computationally expensive. A cooling rate of 1 K/ns is a practical compromise for the simulations. It was shown that, for amorphous iPP, oxygen solubility (*H*_S_^cc,am^) exhibited a nonmonotonic temperature dependence:

- Below 325 K: solubility decreases with temperature, indicating an exothermic process (Δ*H*_S_ < 0) (*T*_g_ = 305.6 K^34^).
- Above 375 K: solubility increases with temperature, indicating an endothermic process (Δ*H*_S_ > 0) (*T*_m_ = 460 K^21^).

This behavior can be explained by the interplay of binding enthalpy (Δ*H*_B_, always negative) and reorganization enthalpy (Δ*H*_R_, always positive) according to van der Vegt.^41^ In amorphous PE, the solubility increased monotonically with temperature, with Δ*H*_S_ = 1.1 ± 0.1 kJ/mol. Lower temperatures could not be studied due to fast crystallization, which hinders direct comparison. Simulation results for iPP show good agreement with some experimental data (e.g., Celina et al. at 400 K^38^) but deviate from those at lower temperatures. Differences in solubility and enthalpy of solvation across studies highlight the influence of crystallinity and thermal history.

Then, semicrystalline samples of iPP were prepared by elongational crystallization and long annealing at 410 K. The samples were then cooled to 298 K in order to perform the solubility measurements. Samples annealed for longer times (up to 5000 ns) develop significant lamellar structures, indicating an ongoing crystallization without reaching a plateau. Moreover, a hexagonal crystal mesophase was found with an edge length equal to 0.6 nm, in accordance with experimental^48^ and computational^47^ observations.

Semicrystalline samples of PE were prepared by both elongational and QC at 365 and 340 K, respectively. The lamellar structure only needed a few nanoseconds to emerge, and further annealing resulted in an increase of the lamellar length. Solubility measurements performed in the same PE semicrystalline samples cooled down at ambient temperature were in line with experimental results by Michaels and Bixler,^1^ which were scaled using the proposed linear relationship by the same authors.

Solubility measurements in the simulated semicrystalline iPP exhibited deviations from the linear relation proposed by Michaels and Bixler,^1^ suggesting either oxygen penetration into the crystalline phase or constraints imposed on the amorphous phase. Longer annealing times, however, reduced this deviation, implying that the development of the lamellar morphology can have an effect on the solubility. Thus, the solubility was correlated with the local structure by characterizing the orientation of the chain segments as quantified through a local order parameter *P*_2,local_. Higher order crystal phases of *P*_2,local_ > 0.8 were impermeable to oxygen, while the solubility of low order crystal phases of 0.6 < *P*_2,local_ < 0.8 decreased with annealing time. The solubility of the amorphous phase was independent of the annealing time for *P*_2,local_ < 0.3. However, the absorption of oxygen in the amorphous phase of high order (0.3 < *P*_2,local_ < 0.6) seemed to be affected by the presence of the lamellar structure.

Our findings imply that further experimental studies are needed to validate simulation predictions across different cooling rates and temperatures, particularly focusing on the effects of crystallinity and thermal history on solubility. Computationally, implementing more sophisticated extrapolation methods, as proposed by Struik,^32,33^ could enhance the accuracy of solubility predictions at realistic cooling rates. Additionally, investigation of a possible transition point of *H*_S_^cc,am^ for PE at lower temperatures and slower cooling rates could provide deeper insights into its glass-transition behavior and solubility properties. Finally, a thorough investigation of the interlamellar phase and how it is affected by annealing time and its correlation to solubility is of great importance.
